# Silencing an ATP-Dependent Caseinolytic Protease Proteolytic Subunit Gene Enhances the Resistance of Rice to *Nilaparvata lugens*

**DOI:** 10.3390/ijms25073699

**Published:** 2024-03-26

**Authors:** Shuting Chen, Miaofen Ye, Peng Kuai, Lin Chen, Yonggen Lou

**Affiliations:** State Key Laboratory of Rice Breeding and Biology & Ministry of Agriculture Key Lab of Molecular Biology of Crop Pathogens and Insects, Key Laboratory of Biology of Crop Pathogens and Insects of Zhejiang Province, Institute of Insect Sciences, Zhejiang University, Hangzhou 310058, China; shtchen@zju.edu.cn (S.C.); 11616070@zju.edu.cn (M.Y.); kpchen7493@163.com (P.K.); chenlin88@yzu.edu.cn (L.C.)

**Keywords:** rice, OsClpP6, *Nilaparvata lugens*, JA, VOCs, defense responses

## Abstract

The ATP-dependent caseinolytic protease (Clp) system has been reported to play an important role in plant growth, development, and defense against pathogens. However, whether the Clp system is involved in plant defense against herbivores remains largely unclear. We explore the role of the Clp system in rice defenses against brown planthopper (BPH) *Nilaparvata lugens* by combining chemical analysis, transcriptome, and molecular analyses, as well as insect bioassays. We found the expression of a rice Clp proteolytic subunit gene, *OsClpP6*, was suppressed by infestation of BPH gravid females and mechanical wounding. Silencing *OsClpP6* enhanced the level of BPH-induced jasmonic acid (JA), JA-isoleucine (JA-Ile), and ABA, which in turn promoted the production of BPH-elicited rice volatiles and increased the resistance of rice to BPH. Field trials showed that silencing *OsClpP6* decreased the population densities of BPH and WBPH. We also observed that silencing *OsClpP6* decreased chlorophyll content in rice leaves at early developmental stages and impaired rice root growth and seed setting rate. These findings demonstrate that an OsClpP6-mediated Clp system in rice was involved in plant growth-defense trade-offs by affecting the biosynthesis of defense-related signaling molecules in chloroplasts. Moreover, rice plants, after recognizing BPH infestation, can enhance rice resistance to BPH by decreasing the Clp system activity. The work might provide a new way to breed rice varieties that are resistant to herbivores.

## 1. Introduction

Herbivores are one of the most important factors that threaten plant fitness. To defend against these threats when they are attacked, plants produce defensive responses. The defense response begins after plants recognize herbivore-associated signals via their receptor complexes; initiation of early signaling events follows, including the influx of calcium ions (Ca^2+^), the burst of reactive oxygen species (ROS), and the activation of mitogen-activated protein kinase (MPK) cascade [1,2,3,4]. The early signaling events activate the signaling pathways mediated by phytohormones, such as jasmonic acid (JA), salicylic acid (SA), ethylene (ET), and abscisic acid (ABA); these pathways ultimately result in extensive changes in transcriptomes and metabolomes of plants via their crosstalk, and these changes enhance the direct and indirect resistance of plants to herbivores [5,6].

The ATP-dependent caseinolytic protease (Clp), also known as the Clp system, is found in the chloroplast stroma and plays a key role in maintaining chloroplast protein homeostasis by degrading misfolded, damaged, superfluous, or unwanted proteins [7,8]. In Arabidopsis, the Clp system consists of a proteolytic core (ClpRP) with both proteolytic and noncatalytic subunits, stabilizing/activating factors, AAA+ (ATPases associated with various cellular activities) chaperone complex, and two adaptors [9]. The ClpRP is composed of two heptameric rings (P and R) and stabilized by plant-specific ClpT1 and ClpT2 proteins. The P ring (formed by a 1:2:3:1 ratio of subunits ClpP3, ClpP4, ClpP5, and ClpP6) has proteolytic activity, while the R ring (formed by a 3:1:1:1:1 ratio of subunits ClpP1, ClpR1, ClpR2, ClpR3, and ClpR4) appears to lack catalytic activity, even though ClpP1 is a proteolytic subunit [10,11]. The chaperone complex composed of ClpC1, ClpC2, and ClpD is attached to the R-ring, and its subunits are capable of triggering conformational changes in a broad range of protein substrates [12,13]. The adaptor protein ClpS1 targets substrate proteins and thereby causes the degradation of the substrate proteins through the prokaryotic N-end rule pathway [9,14]. Metabolomic analysis of plants deficient in the Clp system has indicated that a number of proteins derived from the shikimate metabolism pathway, leucine synthesis pathway, and lipid metabolism pathway may be potential substrates [15]. Currently, only a few of the well-characterized Clp system substrates have been identified in Arabidopsis, such as the glutamyl-tRNA reductase (GluTR) from the tetrapyrrole pathway [16,17] and the deoxyxylulose 5-phosphate synthase (DXS) from the methylerythritol 4-phosphate (MEP) pathway [18,19].

By regulating plastid protein homeostasis, the Clp system is extensively involved in plant embryogenesis, cell growth, and plastid development [20,21]. In *Nicotiana tabacum*, for instance, the knockdown of *ClpP1* retards plant growth and reduces leaf pigmentation [22]. Silencing *ClpR1* in tomatoes causes β-carotene-enriched fruits to become orange instead of red when the fruits are ripe [23]. In rice, the mutation of the gene *OsClpP6*, which encodes the subunit of a Clp system, ClpP6, causes virescent yellow and narrow leaves, reduced height, a small panicle, and increased tiller number in plants [24,25]. Recently, the Clp system has also been reported to play a vital role in plant responses to pathogens. The transcript level of *TaClpS1* in wheat (*Triticum aestivum*), for instance, is significantly induced during infection by *Puccinia striiformis* f. sp. *tritici* (Pst); knockdown of *TaClpS1*, which enhances the resistance of wheat to Pst, is accompanied by an increase in hypersensitive response, the burst of ROS, and the expression of PR genes [26]. However, our knowledge about the role of the Clp system in plant defenses against herbivores is still limited.

Rice is one of the most important food crops worldwide. Brown planthopper (BPH) *Nilaparvata lugens* is a notorious pest on rice and causes yield reductions of up to 30% if not controlled [27]. BPH damages rice by feeding on phloem sap, laying eggs in plant tissues, and transmitting viruses [27]. It has been well documented that BPH infestation activates signaling pathways mediated by a variety of phytohormones, including JA, SA, ET, ABA, and H_2_O_2_ [28,29,30,31,32]; these phytohormone-mediated signaling pathways, especially the JA pathway, play an important role in regulating the production of defensive compounds, and the direct and/or indirect resistance of rice to BPH [30,33,34,35]. Given that the biosynthesis of OPDA, the precursor of JA, from linolenic acid occurs in chloroplasts, it is possible that Clps, as chloroplast proteases, may influence the biosynthesis of JA and thereby influence rice herbivore resistance. However, so far, whether the Clp system is involved in rice herbivore resistance remains unclear.

To explore this issue, we cloned the rice gene *OsClpP6*, which has been reported to encode a Clp proteolytic subunit, Clp6, in rice [24] and investigated its role in the resistance of rice to BPH. By combining chemical analysis, transcriptome, and molecular analyses, as well as insect bioassays, we found that silencing *OsClpP6* enhanced the production of BPH-induced JA, JA-isoleucine (JA-Ile), ABA, and volatile organic compounds (VOCs), as well as the resistance of rice to BPH in both the laboratory and field. However, silencing *OsClpP6* impaired the growth of rice plants. Our findings demonstrate that the OsClpP6-mediated Clp system positively regulates rice growth and development but negatively regulates the resistance of rice to BPH.

## 2. Results

### 2.1. BPH Infestation, Wounding, and MeJA Treatment Suppress the Expression of OsClpP6

The transcriptome data from rice plants infested by gravid females of BPH showed that the transcript level of a Clp proteolytic subunit gene was significantly down-regulated in response to such infestation. By cloning this gene, we found its sequence was 100% identical to that of the previously reported *OsClpP6* [24]. Sequence analysis found that *OsClpP6* includes an open reading frame of 780 bp, which encodes a protein of 260 amino acids (Figure S5).

**Figure 1:**
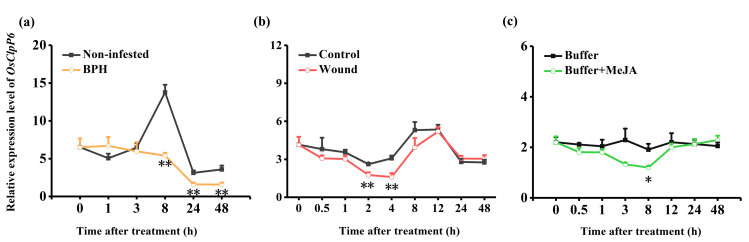
Transcript levels of *OsClpP6* after different treatments. Mean relative expression levels (+SE, *n* = 5) of *OsClpP6* in leaf sheaths of rice plants at different times after they were infested by BPH (**a**), mechanically wounded (**b**), or treated with MeJA (**c**). The asterisks indicate significant differences between treatments and controls (* *p* < 0.05; ** *p* < 0.01, Student’s *t*-tests).

Compared with controls, all of the measured treatments—BPH infestation, mechanical wounding, and MeJA treatment—decreased the transcript levels of *OsClpP6* in rice plants (Figure 1). Notably, BPH infestation suppressed the expression of *OsClpP6* strongly, especially 8 h after BPH infestation when the expression of *OsClpP6* in BPH-infested plants was only 39.2% of that in non-infested plants (Figure 1a). Both mechanical wounding and MeJA treatment only weakly inhibited the expression of *OsClpP6* (Figure 1b,c).

### 2.2. Silencing OsClpP6 Enhances the Resistance of Rice to BPH

**Figure 2:**
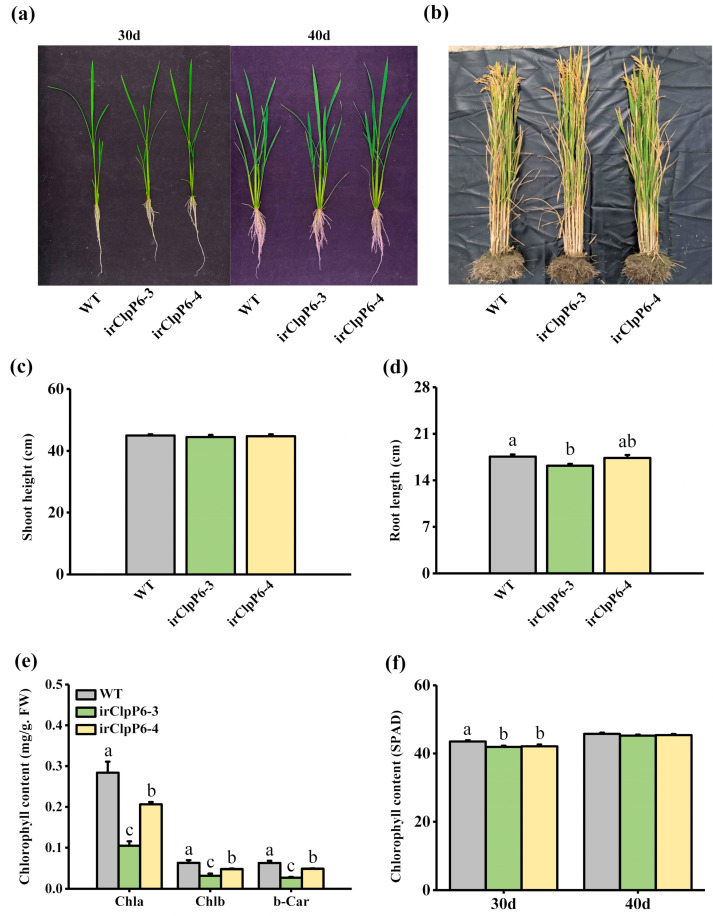
The growth phenotype of WT and irClpP6 lines. (**a**) The growth phenotype of 30-day-old (left) and 40-day-old (right) WT and irClpP6 plants in the greenhouse. (**b**) The growth phenotype of mature WT and irClpP6 plants in the field. (**c**,**d**) Mean shoot height (**c**) and root length (**d**) (+SE, *n* = 10) of 40-day-old WT and irClpP6 plants in the greenhouse. (**e**) Mean content (+SE, *n* = 5) of chlorophyll a (Chla), chlorophyll b (Chlb), and β-carotene (β-Car) in 10-day-old WT and irClpP6 plants. FW, fresh weight, (**f**) mean chlorophyll content (+SE, *n* = 10) in 30-day-old and 40-day-old WT and irClpP6 plants in the greenhouse. Different letters represent significant differences between irClpP6 lines and WT plants (*p* < 0.05, Tukey’s HSD post-hoc tests).

To investigate the function of *OsClpP6* in herbivore-induced defense responses in rice, we obtained two homozygous *OsClpP6*-silenced lines (irClpP6-3 and irClpP6-4) with a single insertion (Figure S3a). Transcriptional analysis showed that the transcript level of *OsClpP6* in the two irClpP6 lines was almost half of that in WT plants 12 h after BPH infestation (Figure S3b). No obvious difference was observed in shoot height between WT and irClpP6 lines (Figure 2a–c). However, silencing *OsClpP6* did reduce the root length of 40-day-old plants slightly (Figure 2d). Moreover, the content of chlorophyll a, chlorophyll b, and β-carotene in irClpP6 seedlings (10 d old) was significantly lower than that in WT plants (Figure 2e). The chlorophyll content of irClpP6 lines at 30 d was still significantly lower than that of WT plants, although there was no difference when plants reached 40 days (Figure 2f).

**Figure 3:**
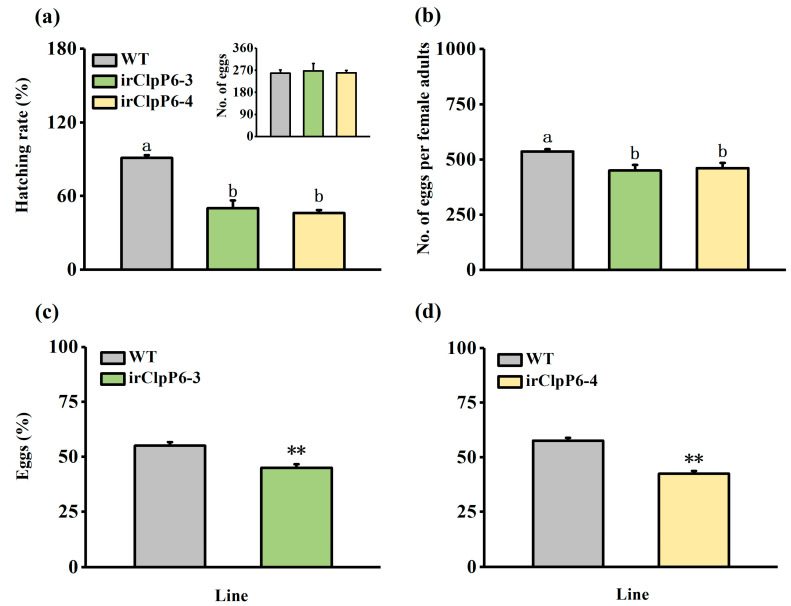
Silencing *OsClpP6* enhances the resistance of rice to BPH. (**a**) Mean hatching rate (+SE, *n* = 10) of BPH eggs on WT and irClpP6 lines. Inserts: mean number of BPH eggs (+SE, *n* = 10) per WT and irClpP6 plant laid by 15 gravid BPH females over 12 h. (**b**) Mean number of eggs (+SE, *n* = 20) per WT and irClpP6 plant laid by one BPH female for 10 d. Different letters represent significant differences between irClpP6 lines and WT plants (*p* < 0.05, Tukey’s HSD post-hoc tests). (**c**,**d**) Mean percentage (+SE, *n* = 10) of BPH eggs per plant on pairs of plants (WT versus irClpP6-3 or irClpP6-4), 48 h after the release of BPH. Asterisks represent significant differences between WT and irClpP6 plants (irClpP6-3 or irClpP6-4) (** *p* < 0.01; Student’s *t*-tests).

Bioassays revealed that the hatching rate of BPH eggs on irClpP6 lines was significantly lower than that of BPH eggs on WT plants, although no difference was observed in the number of eggs laid by 15 gravid BPH females for 12 h between WT and irClpP6 lines (Figure 3a). We also investigated the effect of silencing *OsClpP6* on the fecundity of BPH females and found that the number of eggs laid by a BPH female over 10 d on two irClpP6 lines (about 450) was significantly lower than that laid by a BPH female over 10 d on WT plants (about 536; Figure 3b). Moreover, we observed that gravid BPH females preferred to lay eggs on WT plants rather than on irClpP6 lines: the number of eggs laid on irClpP6-3 and irClpP6-4 plants was only 44.96% and 42.43%, respectively, of that laid on WT plants (Figure 3c,d).

### 2.3. OsClpP6 Negatively Regulates the Expression of Defense-Related Genes in Rice in Response to BPH Infestation

**Figure 4:**
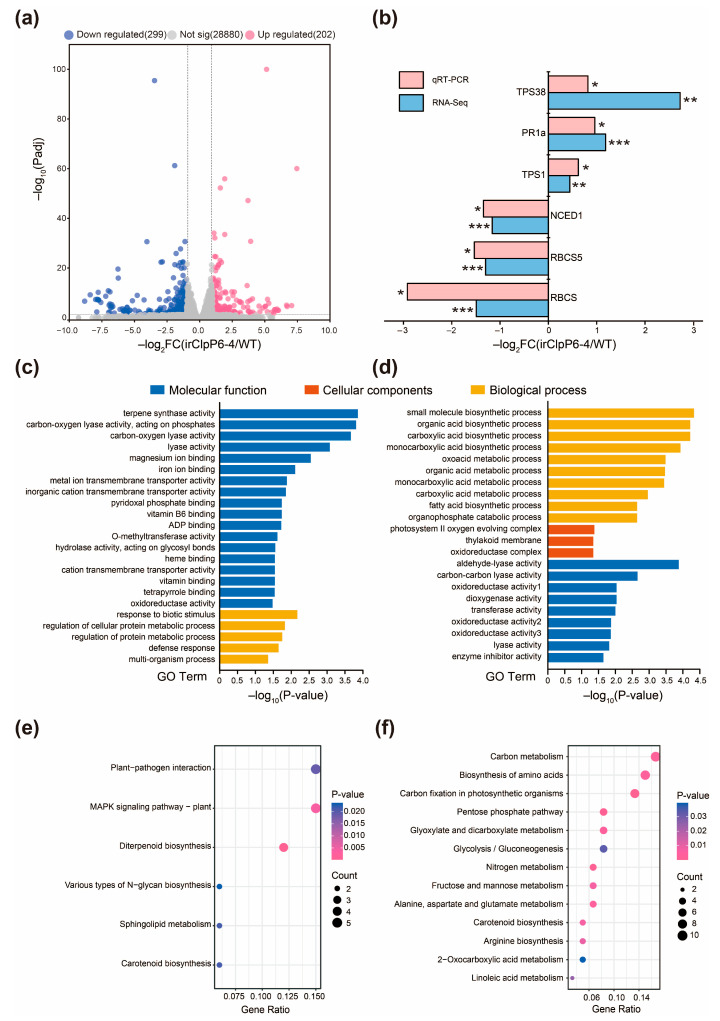
RNA-seq analyses of WT and irClpP6 lines in response to BPH infestation. (**a**) The volcano figure of these differentially expressed genes (DEGs) between irClpP6-4 and WT plants that were infested by BPH for 24 h. (**b**) Verification of DEGs found in transcriptome data by qRT-PCR. The log_2_FoldChange in transcript levels (*n* = 3) of selected DEGs from transcriptome were analyzed by qRT-PCR. Asterisks represent significant differences between irClpP6-4 and WT plants via RNA-Seq (* Padj < 0.05; ** Padj < 0.01; *** Padj < 0.001; FDR) or qRT-PCR (* *p* < 0.05; ** *p* < 0.01; *** *p* < 0.001; Student’s *t*-tests) analysis, respectively. (**c**,**d**) The gene ontology (GO) enrichment analyses of up-regulated (**c**) and down-regulated (**d**) DEGs in irClpP6-4_BPH vs. WT_BPH comparison. The *x*-axis indicates the -log10 transformed *p*-value, and the *y*-axis indicates the name of the GO term description. The bar color indicates the three categories of the terms: molecular functions (blue), cellular components (orange), and biological processes (yellow). (**e**,**f**) The Kyoto Encyclopaedia of Genes and Genomes (KEGG) pathways analysis of up-regulated (**e**) and down-regulated (**f**) DEGs in irClpP6-4_BPH vs. WT_BPH comparison. The gene ratio is the ratio of the number of DEGs annotated in this pathway term to the number of all genes annotated in this pathway term. The dot size indicates the count of DEGs. BPH, BPH-infected plants.

To further clarify the role of OsClpP6 in rice defense against BPH, we used RNA high-throughput sequencing to compare the expression patterns of genes in irClpP6-4 plants with those in WT plants in response to BPH infestation. The RNA-seq data were aligned with those from the rice genome (https://rapdb.dna.affrc.go.jp/download/irgsp1.html, accessed on 7 June 2021). An average of 45 million reads was obtained per sample, where the number of reads aligned to the genome; these and other sequencing data are shown in Table S2. When plants were infested by BPH, 202 genes exhibited higher and 299 genes exhibited lower transcript levels in irClpP6-4 plants than WT plants (Figure 4a, Table S3) (|log_2_FoldChange| > 1; Padj < 0.05; false discovery rate [FDR]). In the absence of BPH infestation, 273 genes had higher and 193 genes had lower transcript levels in irClpP6 plants compared to WT plants (Table S4). In order to verify the authenticity of RNA-Seq, we selected 6 differentially expressed genes (DEGs) and verified the transcriptome results by qRT-PCR. The qRT-PCR results of all 6 DEGs were almost completely consistent with the RNA-seq results (Figure 4b); therefore, the transcriptome data could be used as a reference for the subsequent functional studies of *OsClp6*.

GO analysis indicated that up-regulated genes in the irClpP6-4_BPH plants compared to WT_BPH plants are mainly enriched in the categories molecular functions (which include terpene synthase activity and carbon-oxygen lyase activity) and biological processes (which include responses to biotic stimulus, regulation of protein metabolic process, and defense responses (Figure 4c)); down-regulated genes are highly enriched in biological processes (such as small molecule biosynthesis, organic acid biosynthesis, and other organic syntheses) and molecular functions (including aldehyde-lyase activity and oxidoreductase activity (Figure 4d)). In the absence of BPH infestation, up-regulated genes in irClpP6-4_Con plants compared to WT_Con plants are highly enriched in protein ubiquitination (Table S5), and down-regulated genes are highly enriched in response to oxidative stress (Table S6). Kyoto Encyclopaedia of Genes and Genomes (KEGG) analysis indicated that up-regulated genes in the irClpP6-4_BPH vs. WT_BPH comparison were enriched in plant-pathogen interaction, MAPK signaling pathway, and diterpenoid biosynthesis (Figure 4e); and down-regulated genes were enriched in carbon metabolism, biosynthesis of amino acids, and carbon fixation in photosynthetic organisms (Figure 4f).

### 2.4. Silencing OsClpP6 Enhances the Level of BPH-Induced JA, JA-Ile, and ABA but Reduced the Level of H_2_O_2_

**Figure 5:**
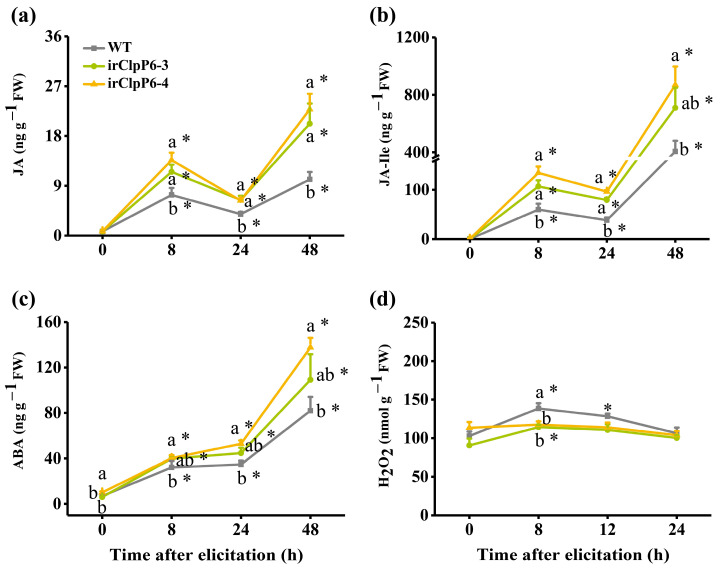
OsClpP6 regulates the biosynthesis of JA, JA-Ile, ABA, and H_2_O_2_ in rice. Mean levels (+SE, *n* = 5) of JA (**a**), JA-Ile (**b**), ABA (**c**), and H_2_O_2_ (**d**) in WT and irClpP6 plants at 0, 8, 12, and 24 h after they were infested by gravid BPH females. Different letters represent significant differences among lines (*p* < 0.05, Tukey’s HSD post-hoc tests). The asterisks indicate significant differences between BPH-infested plants (8, 12, and 24 h) and non-infested plants (0 h) (* *p* < 0.05, Student’s *t*-tests).

The JA-, SA-, ABA-, and H_2_O_2_-mediated signaling pathways play central roles in regulating the resistance of rice to BPH [27,28,36,37]. Therefore, we investigated the content of these signal molecules in WT and irClpP6 lines with or without BPH infestation. As previously reported in [30,31], BPH infestation dramatically induced the production of JA and JA-Ile in both WT and irClpP6 lines. Moreover, the levels of JA and JA-Ile in irClpP6 lines after BPH infestation (for 8, 24, and 48 h) were significantly higher than those in BPH-infested WT plants, whereas no difference was observed in the basal levels of JA and JA-Ile between WT and irClpP6 lines (Figure 5a,b). BPH infestation also significantly enhanced the levels of ABA in both WT and irClpP6 lines. In addition, silencing *OsClpP6* increased the basal and BPH-induced levels of ABA in rice; this result was especially obvious in the irClpP6-4 line (Figure 5c).

BPH infestation induced the production of H_2_O_2_ in WT plants (especially at 8 h after infestation) but not in irClpP6 plants (Figure 5d). Unlike levels of JA and ABA, H_2_O_2_ levels in irClpP6 plants after BPH infestation for 8 h were significantly lower than those in similarly treated WT plants (Figure 5d). Taken together, these results suggest that OsClpP6 regulates the biosynthesis of BPH-induced JA, JA-Ile, ABA, and H_2_O_2_.

### 2.5. OsClpP6 Negatively Modulates the Production of BPH-Induced VOCs in Rice

**Figure 6:**
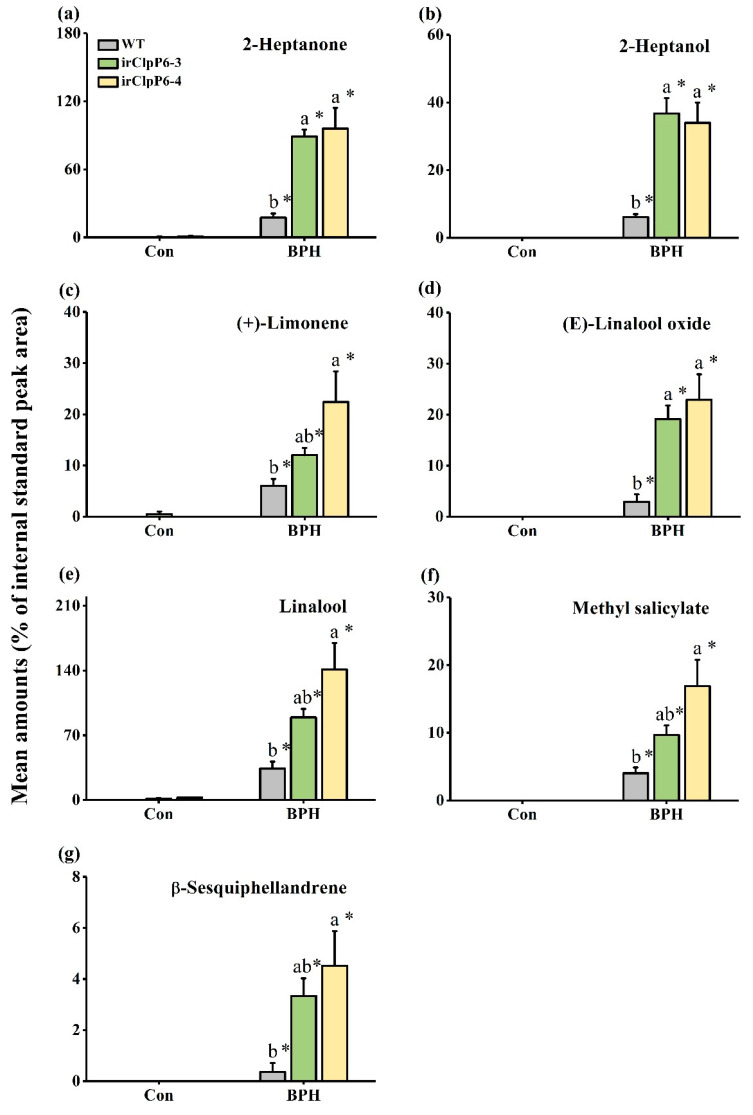
Silencing *OsClpP6* increases the production of BPH-elicited rice volatiles. (**a**–**g**) Mean amounts (% of internal standard peak area) (±SE; *n* = 4–6) of volatile compounds emitted from WT and irClpP6 lines that were infested by gravid BPH females for 24 h or kept non-infested (Con). Different letters represent significant differences among lines (*p* < 0.05, Tukey’s HSD post-hoc tests). The asterisks indicate significant differences between BPH-infested plants (BPH) and non-infested plants (Con) (* *p* < 0.05, Student’s *t*-tests).

Given that the JA-signaling pathways play an important role in the production of herbivore-induced VOCs in plants [30,38,39] and that silencing *OsClpP6* significantly up-regulated the expression of 4 genes involved in terpene biosynthesis (Os12g0491800, Os07g0218200, Os02g0568700, and Os02g0570400) (Table S3), we measured the level of VOCs emitted from WT and irClpP6 lines before and after they had been infested by BPH. The result showed that 7 major VOCs—2-heptanone, 2-heptanol, (+)-limonen, (*E*)-linalool oxide, linalool, methyl salicylate, and β-sesquiphellandrene—were identified in the headspace of WT and irClpP6 lines (Figure 6). Constitutive volatiles emitted from both WT and irClpP6 lines were very low, and no difference was observed between them (Figure 6). BPH infestation significantly promoted the amount of these 7 VOCs emitted from WT and irClpP6 plants, and the levels of these 7 compounds were released in significantly higher amounts from BPH-infested irClpP6 plants than from BPH-infested WT plants (Figure 6a–g).

### 2.6. Silencing OsClpP6 Decreases the Population Densities of BPH and WBPH in the Field

**Figure 7:**
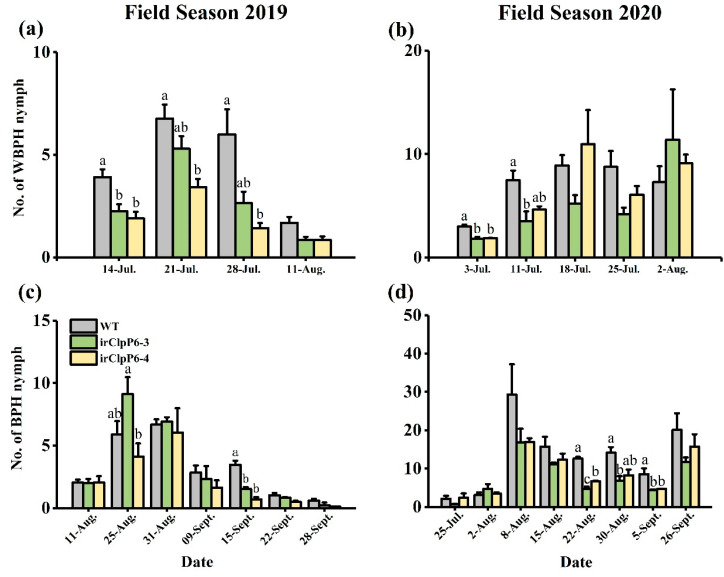
Silencing *OsClpP6* decreases the population densities of BPH and WBPH in the field. Mean number (+SE, *n* = 3) of white-backed planthopper (WBPH) (**a**,**b**) and BPH nymphs (**c**,**d**) per WT and irClpP6 plant in the years 2019 (left panel) and 2020 (right panel). Different letters represent significant differences among lines (*p* < 0.05, Tukey’s HSD post-hoc tests).

To further clarify the ecological function of OsClpP6, a two-year field experiment was carried out in 2019 and 2020. Overall, the population density of both BPH and WBPH was low during the two years, especially in 2019 (Figure 7). Field investigations showed that although silencing *OsClpP6* did not influence the number of WBPH adults (Figure S6a,b), it did significantly decrease the number of WBPH nymphs in the field: the number of WBPH nymphs on the two irClpP6 lines was only 57.29% and 48.34% (on 14 July 2019) and 60.67% and 62.33% (on 3 July 2020), respectively, of that on WT plants (Figure 7a,b). Similarly, no significant difference was observed in the number of BPH adults between WT and irClpP6 lines in 2019 and 2020 (Figure S6c,d); however, the number of BPH nymphs on irClpP6 lines was significantly lower than that on WT plants. The number of BPH nymphs on the two irClpP6 lines was reduced by 44.10% and 19.88% (on 15 Sept. 2019) and 63.2% and 46.6% (on 22 August 2020), respectively, compared with the number of BPH nymphs on WT plants (Figure 7c,d). Silencing *OsClpP6* did not influence the severity of the rice blast (Figure S7a,b), the leaf roll rate of LF (Figure S7c,d), or the number of spiders in the field in both 2019 and 2020 (Figure S8a,b).

In the field plots without pesticides, silencing *OsClpP6* had no significant effect on the number of panicles per plant (Figure S9a), the rate of seed setting (Figure S9b), or the grain yield per plant (Figure S9c), but it did significantly 1000-grain weight in one line (irClpP6-3; Figure S9d). In the field plots under pesticide control, silencing *OsClpP6* also had no significant effect on the panicle number (Figure S9e) or the grain yield per plant (Figure S9g); however, it did significantly reduce the rate of seed sett (Figure S9f). Silencing *OsClpP6* reduced the 1000-grain weight in one line (irClpP6-3) but increased it in the other line (irClpP6-4; Figure S9h). These results indicate that silencing *OsClpP6* has a slight negative effect on the yield of rice in the field.

## 3. Discussion

Chloroplasts are important plant endosymbiotic organelles; they are not only responsible for photosynthesis but also involved in the biosynthesis of many compounds, such as amino acids, fatty acids, nucleotides, secondary metabolites, and phytohormones [10,40]. Recent studies revealed that chloroplast proteases play an important role in plant adaptation to biotic and abiotic stresses by remodeling the chloroplast proteome [7]. Here, we found that the expression of a Clp proteolytic subunit gene in rice, *OsClpP6*, was suppressed by BPH infestation and by wounding. Silencing *OsClpP6* slightly impaired plant growth and development but enhanced levels of BPH-induced JA, JA-Ile, ABA, and VOCs in plants. Finally, silencing *OsClpP6* increased the resistance of rice to BPH in both the laboratory and the field. These results demonstrate that rice plants can enhance their resistance to BPH by regulating the Clp protease system.

Clps have been reported to play an important role in plant growth and development: they are involved in protein turnover and maintain protein homeostasis in chloroplasts. For example, the antisense expression of *ClpP6* in Arabidopsis manifests as chlorosis in young leaves [41]. In tomatoes, ClpR1 regulates both the accumulation of carotenoids and the ripening of tomato fruit [23]. In rice, the mutation of *OsClpR1* results in albino leaves and plant death at the seedling stage and affects the transcript levels of chlorophyll biosynthesis and chloroplast development-related genes [42]. OsClpP6 has been reported to affect the development of rice chloroplasts [24], the growth of leaves, and the mutation of *OsClpP6* in rice (*nal9*), resulting in a phenotype of narrow leaves [25]. Similarly, we found that silencing *OsClpP6* decreased chlorophyll content in 10- and 30-day-old rice. However, silencing *OsClpP6* had no effect on rice leaf morphology, although it decreased the plant’s root length. It has been reported that the influence of ClpP1 on tobacco growth and development is closely related to its level in plants: when the ClpP1 content in plants is reduced by 50%, plants in the early developmental stage grow slowly and have low chlorophyll levels (when they mature, plant growth and chlorophyll levels recover). However, when the ClpP1 content is reduced by 70%, plant leaves become yellow and cannot recover after maturity [22,43]. In our study, the silencing efficiency of *OsClpP6* in irClpP6 plants was lower than 50%, whereas the transcript level of *OsClpP6* in *nal9* was decreased by more than 75% [25]. Therefore, this discrepancy in leaf morphology between our study and the study reported in [25] might be related to the transcript or protein level of OsClpP6 in rice lines (or mutants).

We observed that silencing *OsClpP6* enhanced levels of BPH-induced JA and JA-Ile and of basal and BPH-induced ABA but decreased H_2_O_2_ levels. It has been reported that a phytoene synthase (PSY), which is involved in the carotenoid biosynthesis pathway and closely related to ABA biosynthesis, is a substrate of the Clp system in Arabidopsis [44,45]. Therefore, the increase in levels of basal and BPH-induced ABA in irClpP6 lines might be related to the accumulation of this synthase in chloroplasts. Since JA biosynthesis begins in chloroplasts, silencing *OsClpP6* may also lead to the accumulation of JA biosynthesis-related enzymes, thereby increasing the level of BPH-induced JA and JA-Ile in plants. Moreover, ABA has been reported to promote the production of JA and JA-Ile in rice [46]. Hence, the increase in JA and JA-Ile levels in irClpP6 lines may also be related to high ABA levels in irClpP6 lines. Considering that the JA-signaling pathway negatively regulates the production of H_2_O_2_ in rice [23,24], the decrease in H_2_O_2_ levels in irClpP6 lines infested by BPH compared to in BPH-infested WT plants is probably because of their JA and JA-Ile levels. Further research should elucidate how OsClpP6 regulates the biosynthesis of these defense-related signaling molecules.

In addition to PSY, DXS (deoxyxylulose 5-phosphate synthase), which is the first synthase on the MEP (methylerythritol 4-phosphate) pathway, has also been reported to be a substrate of the Clp proteasome in Arabidopsis [18,19]. The MEP pathway is an important pathway for the biosynthesis of monoterpenes and diterpenes that occur in chloroplasts [47]. Moreover, the JA-signaling pathway plays a central role in regulating the biosynthesis of various kinds of defensive compounds, including VOCs. In rice, for instance, the antisense suppression of the expression of genes related to JA biosynthesis, such as *OsPLDα4*, *OsPLDα5*, *OsAOS1,* and *OsAOS2*, significantly reduced the level of VOCs emitted from plants infested by SSB infestation [29,48]; knocking out *OsAOC* in rice significantly reduced the amount of VOCs, like (+)-limonene and linalool, in plants after BPH infestation [31]. Thus, the increase in BPH-induced rice volatiles in irClpP6 lines compared to WT plants is probably due to the effect of OsClpP6 on enzymes related to the MEP pathway and on the JA-signaling pathway. The specific mechanism by which OsClpP6 affects the production of these VOCs needs to be further investigated. We also investigated the effect of silencing *OsClpP6* on the performance of BPH and WBPH in both the laboratory and field and found that silencing *OsClpP6* significantly reduced the oviposition preference of BPH for plants, the hatching rate of BPH eggs, and the fecundity of BPH females; moreover, it decreased the population density of BPH and WBPH in the field. The JA-signaling pathway in rice has been reported to decrease the hatching rate of BPH eggs [31]. Exogenous application with ABA also enhances rice resistance to BPH, including reducing the number of eggs laid by BPH females and inhibiting the feeding behavior of BPH by promoting the deposition of callose in rice [28,49]. Moreover, the accumulation of H_2_O_2_ in rice decreases the survival of BPH nymphs [28]. Considering that OsClpP6 positively regulates the BPH-induced production of H_2_O_2_, the higher BPH resistance in irClpP6 lines compared to WT plants is probably due to high levels of JA, JA-Ile, and ABA in irClpP6 lines. Plant VOCs influence the behavior and performance of herbivores not only directly but also indirectly, affecting the behavior and performance of natural enemies of herbivores [33,35,50]. S-linalool in rice has been found to attract predators and parasitoids of BPH but to repel BPH females [50]. Methyl salicylate was also reported to repel BPH females but to attract parasitoids [35,51]. Therefore, the reason why BPH female adults preferred to lay eggs on WT plants over irClpP6 plants might be in part related to the higher amount of BPH-induced volatiles (such as S-linalool and MeSA) emitted from irClpP6 lines compared to WT plants. Further research should clarify which defensive compounds that are regulated by JA- and ABA-signaling pathways are involved in the OsClpP6-mediated rice BPH resistance.

We found that, without pesticides, silencing *OsClpP6* had little effect on rice yield in the field, although silencing OsClpP6 did enhance the resistance of rice to rice planthoppers in the laboratory and field. This result is probably related to the low population density of BPH and WBPH in the field. Interestingly, we also observed a significant difference in the 1000-grain weight between the two irClpP6 lines. This discrepancy might be due to the transcript level of *OsClpP6* in rice lines, as stated above. 

## 4. Materials and Methods

### 4.1. Plants and Insects

Rice (*Oryza sativa* L.) genotypes used in this study were Xiushui110 (wild type, WT) and two rice lines silencing *OsClpP*6 (irClpP6 lines, irClpP6-3 and irClpP6-4; these were generated from Xiushui110, see details below). Seedlings were pre-germinated in plastic culture dishes (diameter 5 cm) for 10 days (Figure S1a) and then transferred to a 20 L hydroponic box with rice nutrient solution [52]. After 20–25 days, plants were transferred to 300 mL hydroponic plastic pots individually or in pairs, based on the experiments (Figure S1b). 4 d later, plants were used for experiments. All the plants were grown in a greenhouse with a temperature of 28 ± 2 °C, a photoperiod of 14-h light and 10-h dark, and a relative humidity of around 60%. The BPH population was obtained from rice fields in Hangzhou, China, and maintained on the susceptible rice variety TN1 in a climate chamber at 26 ± 2 °C, with 12-h light photoperiod and 80% relative humidity.

### 4.2. Isolation cDNA of OsClpP6

According to the Rice Genome Annotation Project website (www.rice.plantbiology.msu.edu, accessed on 5 September 2018), full-length cDNA of *OsClpP6* (ID: Os03g0411500) was obtained by PCR amplification. The PCR primers OsClpP6-F (5′-ACTCCTCAGTCCTCGCCTC-3′) and OsClpP6-R (5′-TAGGCTGGCGGCATGTAAAA-3′) were designed using the Primer-BLAST tool (www.ncbi.nlm.nih.gov/tools/primer-blast, accessed on 5 September 2018). The PCR product was cloned into pEASY^®^-Blunt Simple Cloning Vector (Transgen, Beijing, China) and sequenced.

### 4.3. Plant Treatments

For BPH treatment, rice plants were individually covered with glass cages (diameter 4 cm, height 8 cm, with 48 small holes, diameter 0.8 mm) (Figure S1c), into which 15 gravid BPH females were released. Plants with empty glass cages were used as controls. For mechanical wounding, the lower parts of plant shoots were individually pierced 200 times with a needle (no. 3 insect needle; diameter 0.5 mm; Beijing Heli Kechang Technology Development, Beijing, China). Unmanipulated plants were used as controls. For MeJA treatment, plants were grown in a nutrient solution, and then MeJA, which was first dissolved in a small volume of ethanol, was added; the final concentration of MeJA in a nutrient solution was 100 µM. Plants grown in a nutrient solution without MeJA but with the same volume of ethanol were assigned as controls.

### 4.4. Generation and Characterization of Transgenic Plants

For plasmid construction, pCAMBIA1301-RNAi and pHun4c12s CRISPR/Cas9 vectors were used. To construct RNAi recombinant plasmid irClpP6, a 330bp fragment of *OsClpP6* was inserted into the pCAMBIA1301-RNAi vector (Figure S2). The irClpP6 plasmid was transformed into the rice variety Xiushui110 using *Agrobacterium tumefaciens*-mediated transformation. To obtain homozygous T2 lines, GUS staining and southern blot were performed following the method described in [25]. Two homozygous T2 irClpP6 lines (irClpP6-3 and irClpP6-4) with a single insertion (Figure S3) were selected and used in the following experiments.

### 4.5. RNA Extraction and qRT-PCR Analysis

Total RNA extraction was performed using the MiniBEST Plant RNA Extraction Kit (TaKaRa, Dalian, China). For cDNA synthesis, one μg of each total RNA sample was reverse-transcribed using PrimeScript^TM^ RT Master Mix (TaKaRa, Dalian, China), according to the manufacturer’s protocol. The qRT-PCR assay was performed on the CFX96 Real-Time system (Bio-RAD) using TB Green^®^ *Premix* Ex Taq^TM^ (Tli RNaseH Plus) (TaKaRa, Dalian, China). A rice actin gene, *OsACTIN,* served as an internal standard to detect the relative expression levels of target genes. The relative expression level was calculated using the (2^−ΔΔCt^) method. The primer sequences used in qRT-PCR analysis can be found in Table S1. Primer pair efficiency, determined using the standard curve method, was found to be between 90% and 110%. Five biological replications for each line at each time point were performed. 

### 4.6. Determination of Rice Growth and Development Parameters

In this experiment, the length of plant roots, the height of plant shoots, and the chlorophyll content of rice leaves were measured at 30 days and/or 40 days. Plant height was the measurement of the stem base to the longest leaf apex, and root length was the measurement of the stem base to the longest root tip. The content of chlorophyll was determined by SPAD-502 Plus (Konica Minolta Optics, Tokyo, Japan) according to the previous method described in [53]. Ten replications for each line at each time point were performed. The content of chlorophyll a (Chla), chlorophyll b (Chlb), and carotenoids (Car) in leaves of 10-day-old rice plants was also measured using the method described in [54,55]. Briefly, 0.1 g leaves were cut into small pieces, and chlorophyll was extracted with 80% acetone at 4 °C for 24 h in the dark. A Varioskan Flash spectral scanning multimode reader (Thermo Fisher Scientific, Waltham, MA, USA) was used to measure the absorbance of the supernatant at 663 nm (Chla), 645 nm (Chlb), and 470 nm (Car). The content of the 3 compounds was then calculated using the following equations: Chla = (12.21 × D663 − 2.81 × D645) × V/W, Chlb = (20.13 × D645 − 5.03 × D663) × V/W, Car = (1000 × D470 × V/W − 000 × D470 × −000 × D470)/198. Five replications for each line were performed.

### 4.7. Measurement of JA, JA-Ile, SA, ABA, and H_2_O_2_ Content

Plants of WT and irClpP6 lines were randomly assigned to BPH and control treatments. Rice leaf sheaths of plants were harvested at 0, 8, 12, and 24 h after BPH infestation. JA, JA-Ile, SA, and ABA were extracted by ethyl acetate containing labeled internal standards and then analyzed by high-performance liquid chromatography-tandem mass spectrometry (HPLC-MS/MS) according to [56]. For H_2_O_2_ analysis, rice leaf sheaths were collected at 0, 8, 12, and 24 h after BPH infestation. The H_2_O_2_ concentration of each sample was measured using the Amplex Red Hydrogen Peroxide/Peroxidase Assay Kit (Invitrogen, Carlsbad, CA, USA), according to instructions. Five replications for each line at each time point were performed.

### 4.8. Collection, Isolation, and Identification of the VOCs

The collection, isolation, and identification of VOCs were performed following the method described in [33]. Volatiles emitted from individual plants of each line (A1, A30, and WT lines) that were infested with BPH for 24 h or left uninfested were collected. The amounts of compounds were expressed as percentages of their peak areas relative to the peak area of the internal standard (IS, ethyl decanoate) per 8 h of trapping for one plant. Collections were replicated 4–6 times for each treatment. 

### 4.9. BPH Bioassay

To test the impact of silencing *OsClpP6* on the hatching rate of BPH eggs, ten plants of each line (irClpP6-3, irClpP6-4, and WT) were allowed to be infested with 15 gravid BPH females for 12 h. The number of newly hatched nymphs was counted daily until no more nymphs were hatched. Unhatched eggs on each plant were recorded under a microscope, and then the hatching rate of BPH eggs on each line was calculated. 

To investigate the impact of *OsClpP6* on BPH oviposition preference, pots with two plants—an irClpP6-3 plant versus a WT plant, or an irClpP6-4 plant versus a WT plant—were individually confined in the glass cages. Fifteen gravid BPH females were released into each cage. Two days later, insects were removed and the number of eggs on each plant was counted under a microscope. The experiment was replicated 10 times.

To measure the impact of silencing *OsClpP6* on the fecundity of BPH females, plants of WT and irClpP6 lines were used. A pair of BPH adults (one female and one male) that had newly emerged from WT or irClpP6 lines was introduced onto a new plant of the corresponding line (each plant was confined within a glass cage (Figure S1c). After 10 d, the eggs on each plant were counted under a microscope. Twenty replications for each line were performed.

### 4.10. Field Experiments

A two-year field experiment was performed in Changxing, Zhejiang, China (30°53′55″ N, 119°38′37″ E). The experimental plot was divided into nine blocks (6 × 4.5 m), and each block was encompassed by a 0.5 m rice buffer zone (Xiushui110 plants). These blocks were randomly assigned to three lines (two irClpP6 lines and one WT line), each of which underwent three replications (Figure S4). In this experimental plot, no pesticides were sprayed. Rice seeds were sowed in May 2019 or 2020, and seedlings were transplanted to the blocks one month later. The number of nymphs and adults of white-backed planthopper (WBPH) *Sogatella furcifera* and BPH, and the number of spiders, were recorded weekly from June 2019 to September 2019 and from June 2020 to September 2020, using the following method: 15 hills of plants were randomly sampled in each block; for each hill of plants, WBPHs and BPHs on above-ground parts were collected into a plastic tray (length 45 cm × width 33 cm × depth 0.8 cm) by softly tapping plants and then counted. The leaf rolling rate caused by leaf folder (LF) *Cnaphalocrocis medinalis* and the incidence of rice blast *Magnaporthe oryzae* were also estimated based on the overall occurrence of the plot. To measure rice yield, 10 hills of completely mature plants in each block were harvested, and the number of panicles per hill, number of full grains per plant, seed setting rate, and 1000-grain weight were recorded. 

To investigate the possible direct effect of *OsClpP6* itself on rice yield, we performed another field experiment. For this, the experimental plot was again divided into nine blocks (1 × 1 m), and blocks were randomly assigned to the three lines, each line with three replications. In this field experiment, pesticides were applied to control pests and diseases if necessary. During the field experiments in 2019 and 2020, only the population of rice planthoppers (BPH and WBPH) and/or the incidence of rice blasts reached a sufficiently high density or severity at some rice developmental stages that need to be controlled. Therefore, we used 50% pymetrozine (Syngenta, Basel, Switzerland; 450 g per hectare) and 20% tricyclazole (Wenzhou Pesticide Factory, Wenzhou, China; 1500 g per hectare), respectively, to control planthoppers and rice blast. The rice yield was measured using the same method as above. 

### 4.11. RNA-Seq and Transcriptome Analysis

The leaf sheath samples from non-infested (Con) and BPH-infested plants (WT and irClpP6-4 plants) were harvested at 24 h and kept in liquid nitrogen. Leaf-sheaths from 5 plants for each treatment were merged into one sample. Three replications of each treatment were performed. The total RNA integrity was assessed using the RNA Nano 6000 Assay Kit of the Bioanalyzer 2100 system (Agilent Technologies, Santa Clara, CA, USA). The library preparation for transcriptome sequencing was performed by Novogene Co, Ltd. Clean data were obtained by removing reads containing adapter, reads containing ploy-N, and low-quality reads from raw data. The index of the rice reference genome (IRGSP-1.0, https://rapdb.dna.affrc.go.jp/download/irgsp1.html, accessed on 7 June 2021)) was built using Hisat2 v2.0.5, and paired-end clean reads were aligned to the reference genome using Hisat2 v2.0.5. FPKM (fragments per kilobase of exon model per million mapped fragments) was used to estimate gene expression levels. Differential expression analysis of two groups (irClpP6-4_BPH vs. WT_BPH and irClpP6-4_Con vs. WT_Con) was performed using the DESeq2 R package (1.20.0) (Padj < 0.05 and |log_2_FoldChange| > 1).

To validate the transcriptome data, six DEGs (irClpP6-4_BPH vs. WT_BPH group) were selected to test their relative transcript levels in corresponding samples that were used for RNA-seq by qRT-PCR, following the method described in Section 4.5.

Gene Ontology (GO) and Kyoto Encyclopaedia of Genes and Genomes (KEGG) enrichment analysis of differentially expressed genes was implemented by the clusterProfiler R package. Using TB-tools software, v0.665, we generated the diagram that shows enriched GO terms.

### 4.12. Data Analysis

Two treatment data were analyzed using Student’s *t*-tests. Data from three or more treatment groups were analyzed using one-way ANOVA; if the ANOVA was significant (*p* < 0.05), then it was followed by Tukey’s HSD post-doc test. All tests were carried out with IBM SPSS Statistics 24.0. Data were log or square root transformed to meet requirements for the homogeneity of variance.

## 5. Conclusions

We demonstrate that OsClpP6, a Clp system proteolytic subunit, is a positive modulator of rice growth and development. In addition, the expression of *OsClpP6* is suppressed by BPH infestation and negatively regulates the resistance of rice to BPH by influencing both JA- and ABA-signaling pathways. These findings provide a compelling example of how plants, after recognizing herbivore infestation, enhance their resistance to herbivores by altering a Clp protease system; this change resulted in the variation of the biosynthesis of biologically active compounds, such as JA, JA-Ile, and ABA. Moreover, the results reveal a new mechanism that plants resist herbivores, which may be used for breeding crop varieties resistant to herbivores in the future.

## Data Availability

The data of this study are available from the corresponding author, [Y.L.], upon request.

## Supplementary Materials

The following supporting information can be downloaded at: https://www.mdpi.com/article/10.3390/ijms25073699/s1.
