# Supplementary material for: Silencing an ATP-Dependent Caseinolytic Protease Proteolytic Subunit Gene Enhances the Resistance of Rice to Nilaparvata lugens
Source: Int J Mol Sci. 2024 Mar 26;25(7):3699. doi: 10.3390/ijms25073699 (PMC11011769; doi:10.3390/ijms25073699)
Supplement: Supplementary file 1 [file ijms-25-03699-s001.zip › ijms-ClpP6-SI figures-0228.pdf]

Supporting Information

Silencing an ATP-dependent caseinolytic protease proteolytic subunit gene enhances the resistance of rice to *Nilaparvata lugens*

Shuting Chen, Miaofen Ye, Peng Kuai, Lin Chen, Yonggen Lou*

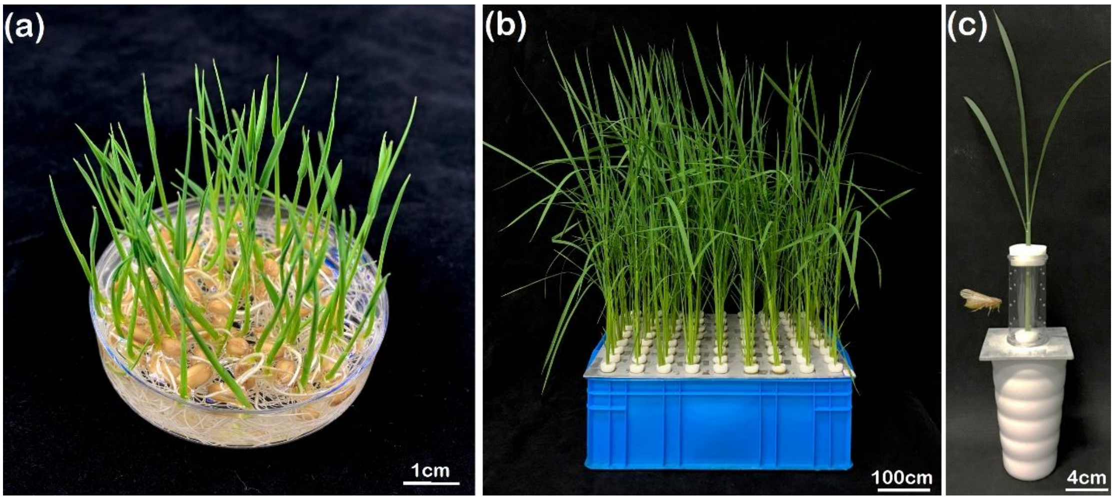

Figure S1. Setups for herbivore bioassay or treatment.

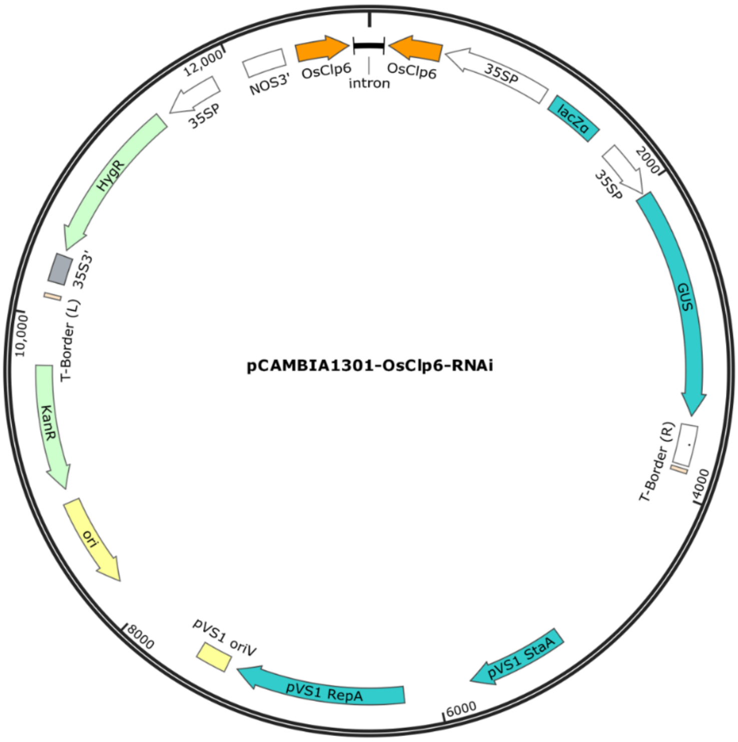

Figure S2. Vectors used in this study. The vector used for obtaining transgenic plants by silencing *OsClpP6*.

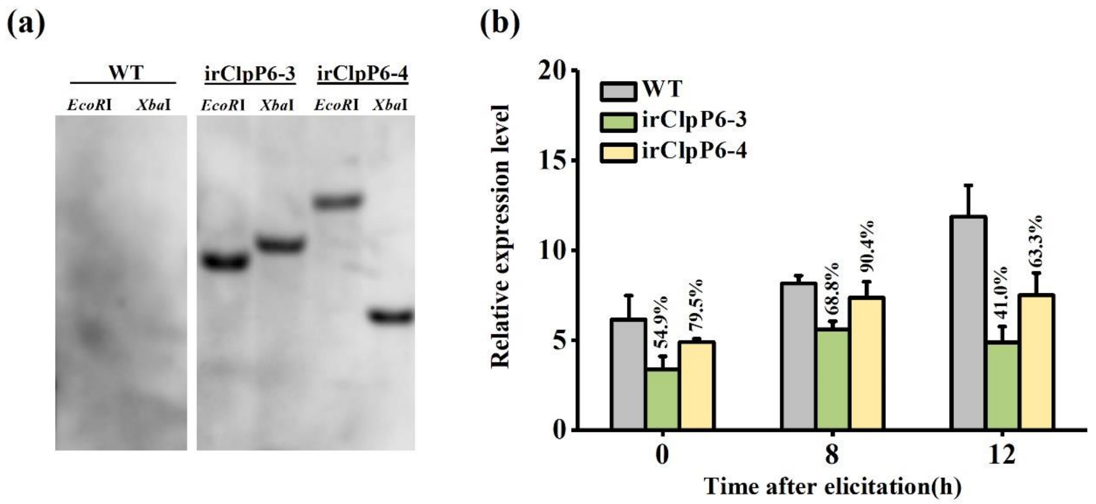

Figure S3. DNA gel-bolt analysis of WT and irClpP6 lines and the transcript levels of *OsClpP6* in irClpP6 lines. (a) Genomic DNA was digested with EcoRI or XbaI. The blot was hybridized with a probe specific for reporter gene GUS. (b) Mean expression levels (+ SE, n = 5) of *OsClpP6* in WT and irClpP6 lines at 0, 8 h and 12h after BPH infestation.

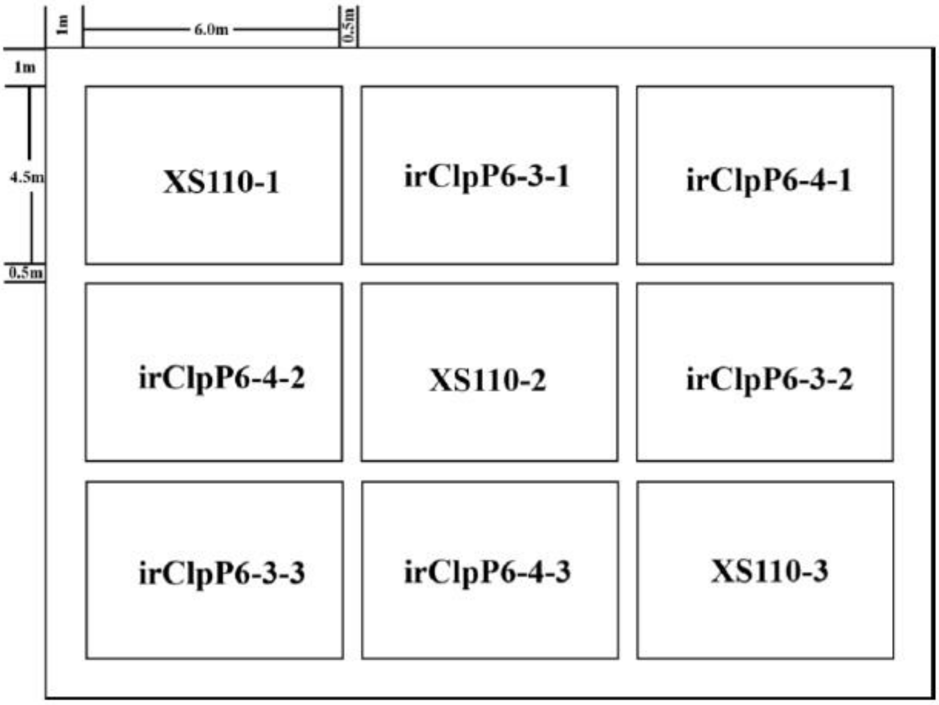

Figure S4. Diagram of field experiment for OsClpP6.

```
1         atggcgcctatggccatctccaccccgctcgccctccgcgcctccccgacccgcctcctc
1          M  A  P  M  A  I  S  T  P  L  A  L  R  A  S  P  T  R  L  L

61        tcccgcaggcggagcggagccaaatcaggcgtggctctcccaggtccacaatttgtacca
21         S  R  R  R  S  G  A  K  S  G  V  A  L  P  G  P  Q  F  V  P

121       cctggtatttcttcaaagttggacgagaggatacattgtcattcttctctgaggaaaaat
41         P  G  I  S  S  K  L  D  E  R  I  H  C  H  S  S  L  R  K  N

181       acaattgtagcatcagagaatgaaaatccacctttaatgcctgccataatgactcctgct
61         T  I  V  A  S  E  N  E  N  P  P  L  M  P  A  I  M  T  P  A

241       ggtgctcttgatctggcaactgtattgttggggaaccgcattatcttcattggtcaatat
81         G  A  L  D  L  A  T  V  L  L  G  N  R  I  I  F  I  G  Q  Y

301       attaactcgcaagtagcacagcgtgtaatatcacagcttgtcacacttgctgctgttgat
101        I  N  S  Q  V  A  Q  R  V  I  S  Q  L  V  T  L  A  A  V  D

361       gaagaggctgatattctgatctacctgaactgccccggcggaagtctctactccatctta
121        E  E  A  D  I  L  I  Y  L  N  C  P  G  G  S  L  Y  S  I  L

421       gcaatttatgattgcatgtcctggatcaagcccaaagttggaacagtgtgctttggtgtt
141        A  I  Y  D  C  M  S  W  I  K  P  K  V  G  T  V  C  F  G  V

481       gttgctagccaggcagcaattatacttgctggcggtgagaagggaatgcgttatgccatg
161        V  A  S  Q  A  A  I  I  L  A  G  G  E  K  G  M  R  Y  A  M

541       ccaaatgctagagtaatgattcatcaacctcaaggtgtatcagagggtaatgtggaggag
181        P  N  A  R  V  M  I  H  Q  P  Q  G  V  S  E  G  N  V  E  E

601       gtgaggcgacaggttggggaaaccatttatgctcgtgataaagttgataagatgtttgct
201        V  R  R  Q  V  G  E  T  I  Y  A  R  D  K  V  D  K  M  F  A

661       gcttttactgggcaaaccttggatatggtacaacagtggacagagagggatcgtttcatg
221        A  F  T  G  Q  T  L  D  M  V  Q  Q  W  T  E  R  D  R  F  M

721       tcttcatctgaagccatggactttggactagttgatgccctgctggaaacaagatactaa
241        S  S  S  E  A  M  D  F  G  L  V  D  A  L  L  E  T  R  Y  *
```

Figure S5. Sequences of nucleotides and deduced amino acids of OsClpP6.

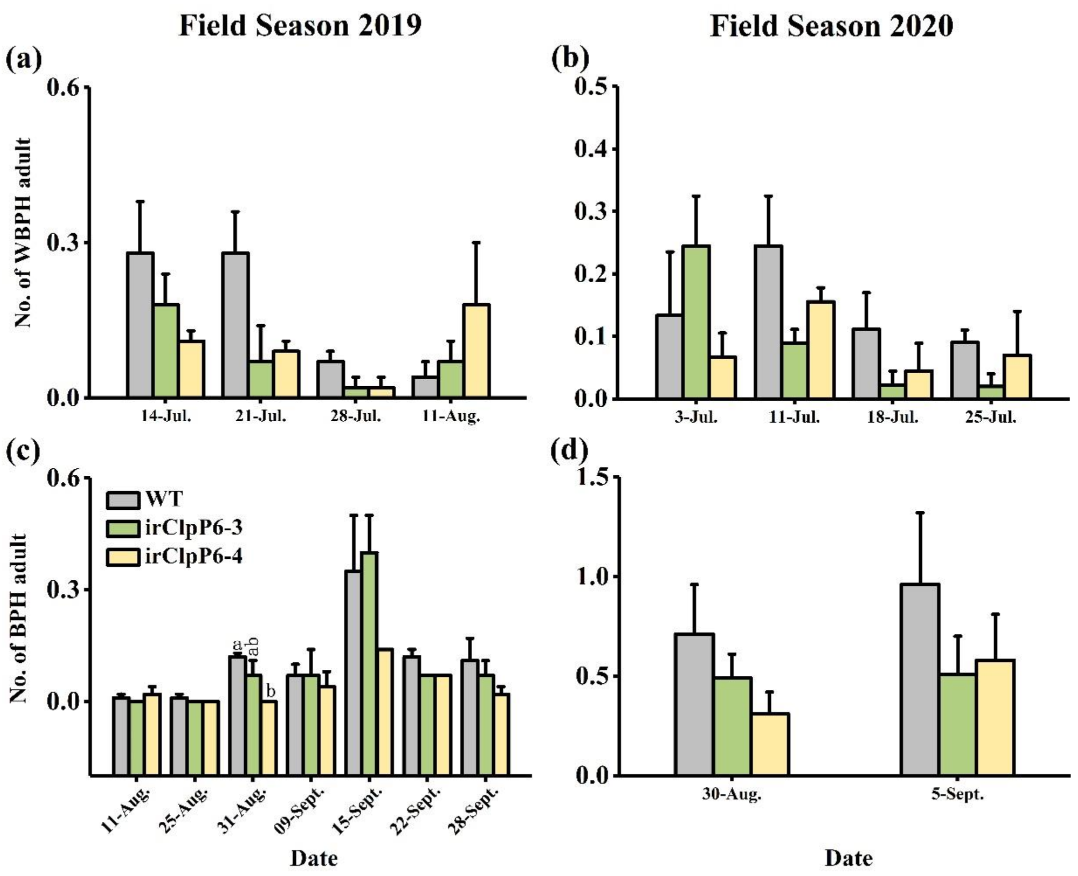

Figure S6. Silencing *OsClpP6* decreases the population densities of WBPH and BPH adults in the field. Mean number (+ SE, $n = 3$) of white-backed planthopper (WBPH) (a-b) and BPH adults (c-d) per WT and irClpP6 plant in year 2019 (left panel) and year 2020 (right panel). Different letters represent significant difference among lines ($p < 0.05$, Tukey's HSD post-hoc tests).

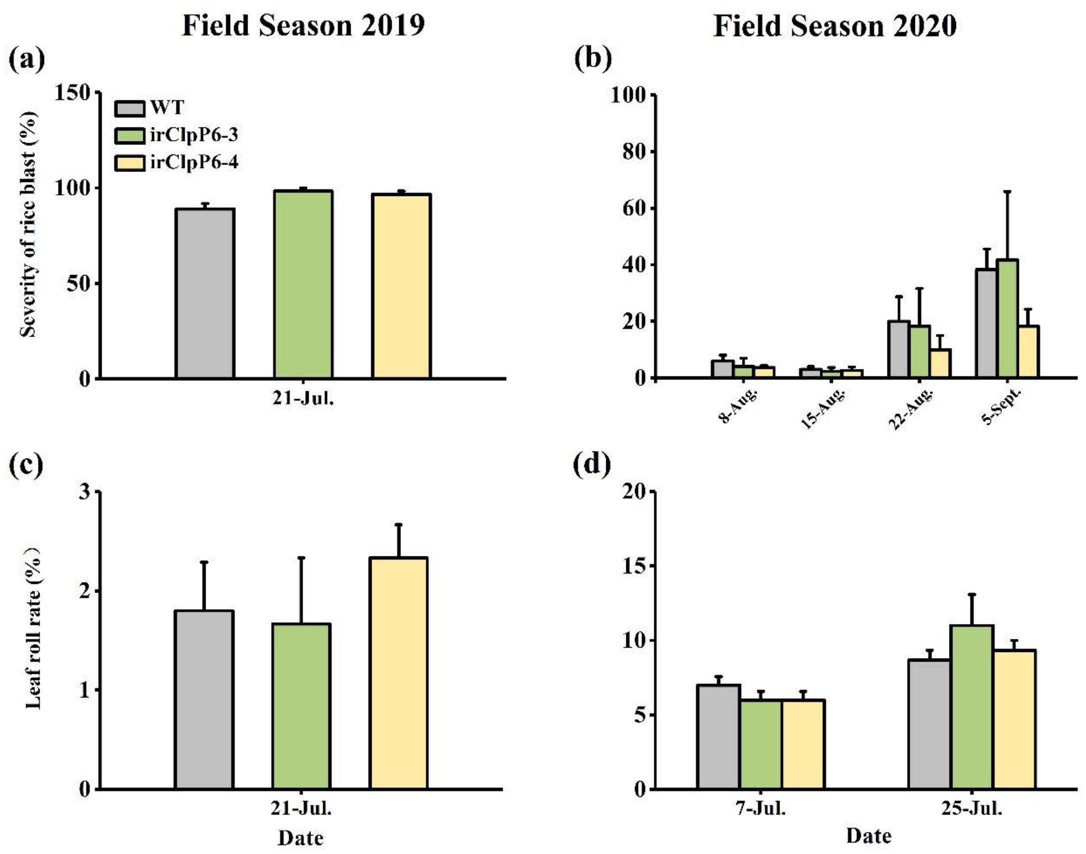

Figure S7. OsClpP6 does not affect other diseases and pests. (a-d) Mean severities (+ SE, *n* = 3) of rice blast disease (a-b) and mean leaf roll rate (+ SE, *n* = 3) caused by *Cnaphalocrocis medinalis* (c-d) per WT and irClpP6 plant in year 2019 (left panel) and year 2020 (left panel).

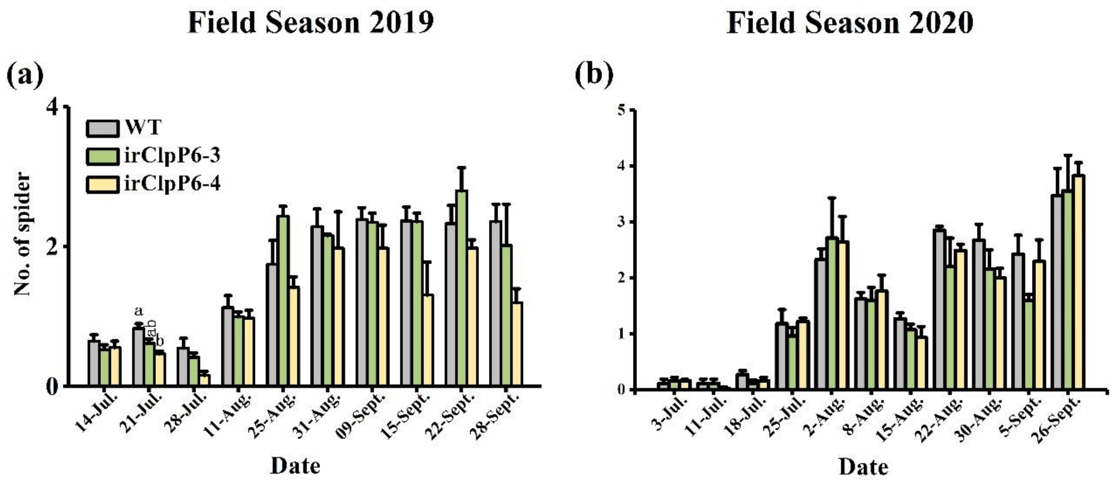

Figure S8. Silencing *OsClpP6* has little effect on natural enemies. Mean number (+ SE, n = 3) of spiders per WT and irClpP6 plant in year 2019 (a) and year 2020 (b). Different letters represent significant difference among lines ($p < 0.05$, Tukey's HSD post-hoc tests).

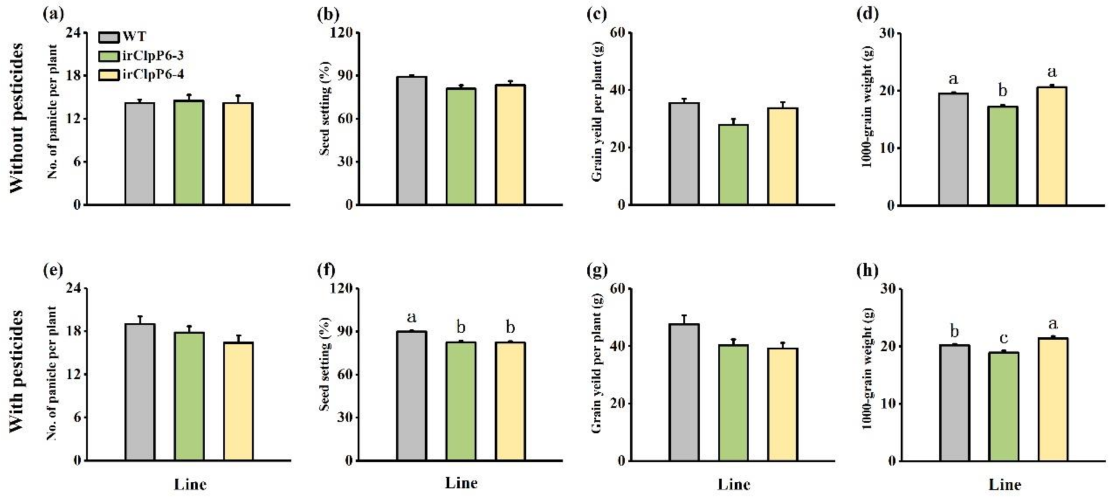

Figure S9. The effect of OsClpP6 on the yield of rice. Mean number of panicle per plant (a, e), seed setting rate (b, f), grain yield per plant (c, g) and 1000-grain weight (d, h) (+ SE, $n = 3$) of WT and irClpP6 lines in 2020 in the field without pesticides (up panel) and with pesticides (down panel). Different letters represent significant difference among lines ($p < 0.05$, Tukey's HSD post-hoc tests).
